# The impact of exercise intensity and duration for swim training‐induced adaptations in cardiac structure and function in women with mild hypertension

**DOI:** 10.14814/phy2.70116

**Published:** 2024-11-01

**Authors:** Tórur Sjúrðarson, Nikolai B. Nordsborg, Jacobina Kristiansen, Lars Juel Andersen, Peter Krustrup, Kasper Kyhl, Magni Mohr

**Affiliations:** ^1^ Faculty of Health Sciences, Centre of Health Science University of the Faroe Islands Tórshavn Faroe Islands Denmark; ^2^ Department of Nutrition, Exercise and Sports University of Copenhagen Copenhagen Denmark; ^3^ Department of Medicine National Hospital of the Faroe Islands Torshavn Faroe Islands Denmark; ^4^ Department of Cardiology Aarhus University Hospital Aarhus Denmark; ^5^ Department of Cardiology Zealand University Hospital Roskilde Denmark; ^6^ Department of Sports Science and Clinical Biomechanics, SDU Sport and Health Sciences Cluster (SHSC) University of Southern Denmark Odense M Denmark; ^7^ Danish Institute for Advanced Study (DIAS) University of Southern Denmark Odense M Denmark; ^8^ Department of Cardiology Copenhagen University Hospital Rigshospitalet Copenhagen Denmark

**Keywords:** cardiac adaptation, cardiac function, cardiac structure, echocardiography, swimming

## Abstract

This study aimed to investigate the impact of swim training intensity and duration on cardiac structure and function in mildly hypertensive women. Sixty‐two mildly hypertensive women were randomized to 15 weeks of either (1) high‐intensity swimming (HIS, *n* = 21), (2) moderate‐intensity swimming (MOD, *n* = 21) or (3) control (CON, *n* = 20). Training sessions occurred three times per week. Cardiac measurements were conducted using echocardiography pre‐ and post‐intervention. Both the HIS and MOD groups demonstrated significant within‐group increases in left ventricular mass: 7.3% [1.2; 13.2] (*p* = 0.02) for HIS and 6.2% [0.5; 11.8] (*p* = 0.03) for MOD. The MOD group also demonstrated a significant increase in left ventricular internal dimension at end‐diastole by 2.4% [0.2; 4.6] (*p* = 0.03). Post‐hoc analysis of diastolic function markers revealed reduced mitral valve A velocity in both HIS (−14% [−25; −3], *p* = 0.02) and MOD (−13% [−23; −3], *p* = 0.01), leading to increased mitral valve E/A ratios of 27% [10; 47] (*p* = 0.003) and 22% [5; 40] (*p* = 0.01), respectively. Additionally, only MOD demonstrated increased left atrial diameter of 4.9% [0.7; 9.1] (*p* =0.02). A significant time×group effect (*p* = 0.02) existed for global longitudinal strain, which increased by 1.6% [0.2; 3.0] (*p* = 0.03) in MOD only. In conclusion, swim training for 15 weeks increased left ventricular mass and improved markers of diastolic function in mildly hypertensive women. These independent of exercise intensity and duration in mildly hypertensive women.

## 1 INTRODUCTION

Arterial hypertension (HTN) remains a primary modifiable risk factor for cardiovascular mortality and morbidity, encompassing a well‐established spectrum of complications including stroke, coronary artery disease, atrial fibrillation, renal insufficiency, and heart failure (Lewington et al., 2002). Indeed, hypertension can provoke a chain of pathological changes in the myocardium such as the development of left ventricular (LV) hypertrophy, fibrosis and thereby stiffening of the ventricle. A healthy lifestyle with regular physical exercise is recommended as part of the non‐pharmacological treatment of hypertension (Andersen et al., 2014; Cornelissen et al., 2010; Cornelissen & Fagard, 2005; Fagard & Cornelissen, 2007). However, there is growing recognition that different sports modalities elicit different cardiac stimuli leading to distinct LV adaptations (Spirito et al., 1994; Venckunas, Lionikas, et al., 2008; Venckunas, Raugaliene, et al., 2008; Wasfy et al., 2015), but the optimal exercise modalities for inducing health‐beneficial cardiac remodeling remains undefined.

Compared to conventional endurance exercises, swimming presents an appealing form of exercise training for diverse patient populations requiring a high‐intensity exercise training stimuli, because it allows for regular high‐intensity training with minimum risk of musculoskeletal complications due to its low weight‐bearing nature. Swimming is performed in a prone or supine position and includes water submersion, and, according to experimental models, these unique physiological factors contribute to augmented central venous return and cardiac preload (Epstein, 1978; Greenleaf et al., 1980; Holmér, 1979; Nielsen, 1983), thus increasing cardiac volume stress. Consistent with the notion that swimming induces a mainly isotonic, or volume‐loading cardiac stress, Wasfy et al. (2019) demonstrated that a 12‐week period of intensified swim training efficiently stimulated eccentric left‐ventricular remodeling characterized by a relatively larger dilation of LV cavity than LV wall thickening, and concomitant improvements in markers of resting LV systolic‐ and diastolic function in competitive collegiate swimmers. However, these findings cannot be translated directly to the effect of swim training in sedentary individuals, and the effect of swimming on cardiac remodeling in sedentary, hypertensive individuals remains largely undetermined.

Therefore, the purpose of the present study was to extend our knowledge of swimming‐induced cardiac remodeling in trained swimmers to a sedentary, hypertensive population. Specifically, we investigated the effects of a 15‐week high‐intensity swimming protocol and a moderate‐intensity swimming protocol on cardiac structure and function in mildly hypertensive middle‐aged women using echocardiography. We hypothesized that the two exercise interventions would induce different cardiac adaptations due to marked differences in cardiac stimulation.

## 2 MATERIALS AND METHODS

### 2.1 Compliance with ethical standards

The study protocol was approved by the ethical committee of the Faroe Islands and conducted in accordance with the Declaration of Helsinki (1964). The participants provided written informed consent after receiving written and oral information of the study protocol.

### 2.2 Participants

Eighty‐three women were enrolled in the study based on the inclusion criteria of mild hypertension (an office systolic blood pressure [SBP] between 130 and d159 mmHg and/or a diastolic blood pressure (DBP) between 85 and 99 mmHg (Williams et al., 2018)), BMI ≥ 25 kg/m^2^, premenopausal and at least 2 years of a sedentary lifestyle. Premenopausal status was determined based on self‐reported regular menstrual cycles within the past 12 months and the absence of menopausal symptoms. Hormonal assays (e.g., estrogen, FSH levels) were not conducted, which we recognize may have resulted in the inclusion of participants in perimenopausal transition. A total of 62 women (see Table 1 for participant characteristics) were enrolled in the present study and 21 were randomly assigned to a football group, which is part of another study (Mohr, Lindenskov, et al., 2014; Sjúrðarson et al., 2024).

**TABLE 1:** Participant characteristics.

Outcome	HIS	MOD	CON
Age (years)	44.1 ± 4.7	46.9 ± 3.0	43.7 ± 4.3
Height (cm)	164 ± 6	165 ± 5	166 ± 6
Weight (kg)	77.1 ± 8.7	84.2 ± 19.1	77.3 ± 10.1
BMI	28.8 ± 3.7	30.7 ± 5.7	28.0 ± 4.1
SBP (mmHg)	138 ± 18	142 ± 18	134 ± 18
DBP (mmHg)	86 ± 14	87 ± 9	82 ± 9
MAP (mmHg)	103 ± 18	105 ± 18	99 ± 2
RHR (bpm)	76 ± 9	78 ± 12	77 ± 7

*Note*: Values are presented as means ± standard deviation in participants allocated to HIS, MOD and CON.

Abbreviations: BMI, body mass index; DBP, diastolic blood pressure; MAP, mean arterial pressure; RHR, resting heart rate; SBP, systolic blood pressure.

### 2.3 Experimental design

The study was designed as a randomized, controlled trial. Parts of the acquired data have been published previously (Mohr, Nordsborg, et al., 2014; Nordsborg et al., 2015; Sjúrðarson et al., 2024), including detailed analyses of blood pressure and resting heart rate measurements, as reported by Mohr et al. (2014). Participants were randomly allocated into either (1) a high‐intensity intermittent swimming group (HIS, *n* = 21), (2) a moderate‐intensity continuous swimming group (MOD, *n* = 21) or (3) a control group (CON, *n* = 20) (Table 1). HIS and MOD were prescribed three weekly exercise training sessions for 15 weeks, whereas CON was explicitly instructed to maintain habitual activity levels at the same level as before enrolment in the study. Echocardiographic scans were completed for each participant within 10 days preceding the start of the intervention and 48–72 h following the conclusion of the final training session. Data pertaining to dietary intake and menstrual cycle were not collected, and hence, not controlled for in the present study.

### 2.4 Training intervention

HIS completed an average of 44 ± 4 training sessions during the 15‐week intervention period, each lasting approximately 15–25 min (3–5 min of effective swimming). HIS sessions comprised 6–10 × 30‐s all‐out front‐crawl intervals interspersed with 2 min of passive recovery in accordance with established training principles (Gibala & McGee, 2008). Participants allocated to HIS underwent a ramp‐up phase consisting of six intervals in Weeks 1–6, increasing to eight intervals in Weeks 7–12 and reaching 10 intervals per session in the final 3 weeks.

MOD completed an average of 43 ± 4 training sessions over the same period, each lasting 1 h and comprising continuous front‐crawl. During each MOD session, participants were explicitly told to cover as much distance as possible. Trained swimming coaches were present during all training sessions to give technical advice and control the intensity and duration of the training. Heart rate loading (Polar Vantage NV chest belt monitor, Polar Electro Oy, Kempele, Finland) was measured in a session in Week 1 and 15 and swim‐distance was registered in every session (see also Mohr et al. (2014)) to record exercise intensity. HR_mean_ and HR_peak_ during the HIS intervention were 85.5 ± 1.1 and 95.3 ± 1.1% HR_max_, respectively, which was higher (*p* < 0.05) than MOD (72.5 ± 0.9 and 79.1 ± 1.0% HR_max_). HR_max_ was determined with a Yo‐Yo Intermittent Endurance Test, level 1. Swim distance per swimming interval increased (*p* < 0.05) by 28 ± 6% from Week 1 to 15 in HIS, while in MOD the swim distance per session increased (*p* < 0.05) by 52.8 ± 3.2%.

### 2.5 Blood pressure measurements

Blood pressure measurements were conducted at the hospital following an overnight fast. Briefly, participants rested in a supine position for 2 h. During this period, systolic blood pressure (SBP) and diastolic blood pressure (DBP) were recorded every 30 min using a validated automatic blood pressure monitor (HEM‐709; OMRON, IL, USA). Four readings were taken in total, and the average was used as the final blood pressure value. The mean arterial pressure (MAP) was calculated using the formula: MAP = (SBP/3) + (2 × DBP/3). However, the results from the blood pressure measurement have been reported elsewhere (Mohr, Nordsborg, et al., 2014).

### 2.6 Echocardiography

**FIGURE 1:**
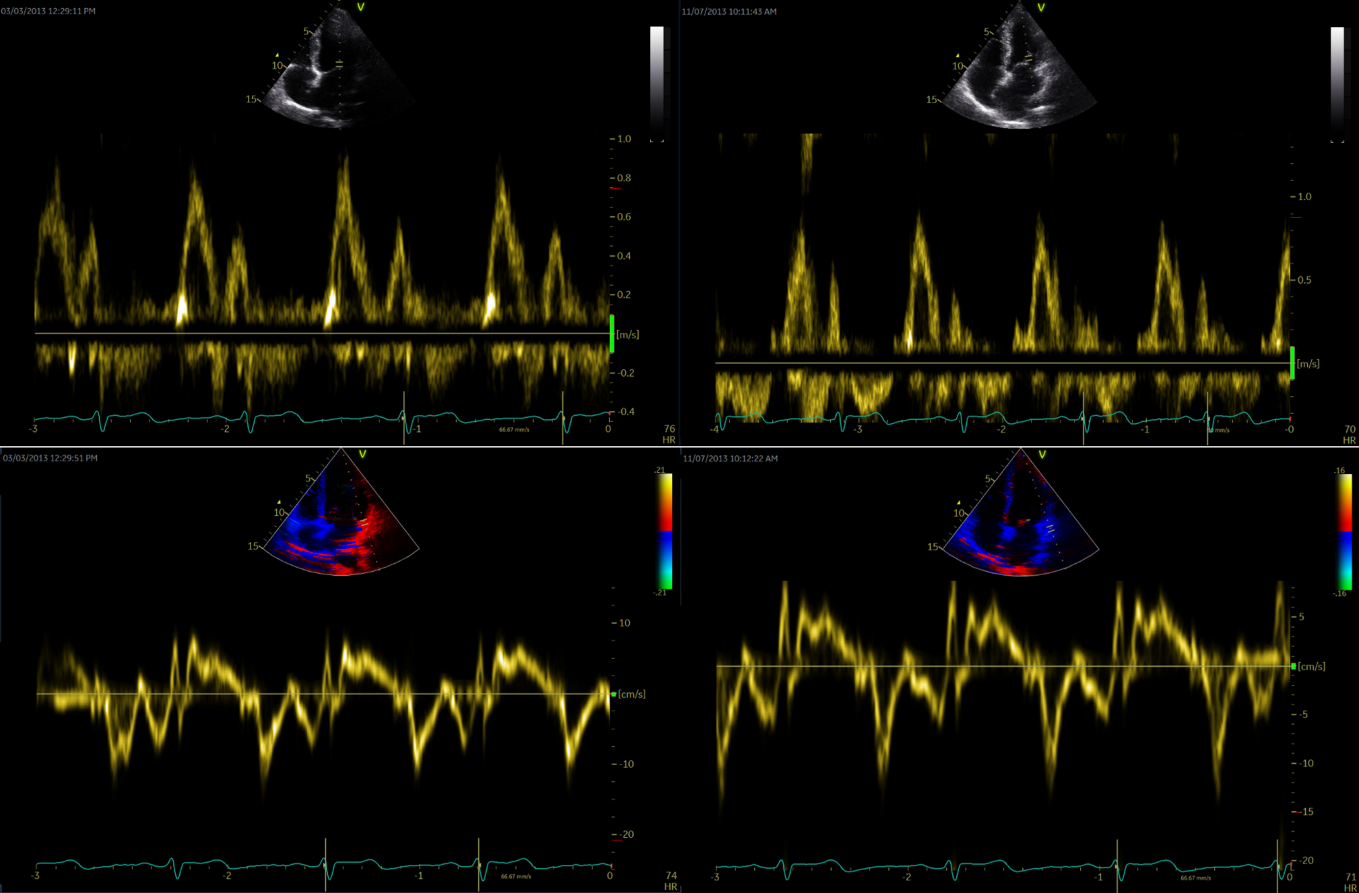
The figure shows examples of representative original Doppler echocardiographic images taken pre‐ and post‐intervention in a participant from HIS. Top: The pulsed Doppler recordings of mitral inflow pre (left) and post (right) intervention, showing early (E wave) and late (A wave) diastolic transmitral flow. Bottom: Pulsed‐wave tissue Doppler measurement at the lateral mitral annulus pre (left) and post (right) intervention, used to assess E′.

Echocardiography was conducted by a cardiologist blinded to trial groups on a GE Vivid E9 ultrasound (GE Medical System, Horten, Norway) using a 2.5 MHz transducer and participants in the left lateral supine position. Two‐dimensional (2D), M‐mode, and Doppler imaging techniques were used to create the images. The analysis was conducted in a blinded manner using the EchoPac software (GE Medical System, Norway). The left ventricle (LV) and right ventricle (RV) dimensions, interventricular septum thickness, and left atrium (LA) diameter were measured in parasternal long‐axis 2D recordings. Using Simpson's biplane approach, LV volumes and ejection fraction were calculated (Schiller et al., 1989). In the apical four‐chamber view, pulsed Doppler measurements of mitral inflow yielded peak transmitral flow in early diastole (E) and during LA contraction (A), E/A ratio, and deceleration time. Average early diastolic mitral annulus velocity (E′) was measured in both the medial and lateral myocardial wall using pulsed‐wave tissue Doppler imaging (Sjúrðarson et al., 2022). RV systolic function was assessed using M‐mode to measure TAPSE, defined as the total displacement of the tricuspid annulus in the longitudinal direction from end‐diastole to end‐systole. LV global longitudinal strain was obtained by 2D image speckle tracking analysis as performed previously (Leitman et al., 2004; Reisner et al., 2004). Indexed values were normalized for body surface area (BSA) using the Mosteller equation. Representative echocardiography images are displayed in Figure 1.

### 2.7 Statistical analysis

Baseline characteristics are presented as means with standard deviation and compared using a one‐way ANOVA. Echocardiographic data are presented as means with 95% confidence intervals, and analyzed using linear mixed method for repeated measures using SPSS (IBM SPSS Statistics, version 28.0.0). The model included time (pre vs. post), trial groups (HIS, MOD, and CON) and a time×group interaction effect as fixed effects. The interaction term was used to evaluate whether the trial groups showed different responses in the obtained cardiac variables from pre to post intervention. A Sidak‐adjusted pairwise comparison was conducted for all obtained cardiac end points to determine potential within‐group time‐specific differences and between‐group differences at baseline and post‐intervention. Random variation and repeated effects were defined from participants. Model fit was assessed by graphical methods. Finally, an independent samples *t*‐test was used to evaluate potential between‐group differences in mean and peak heart rate during HIS and MOD. Statistical tests were two‐sided, and the level of significance was set to 0.05.

## 3 RESULTS

### 3.1 Baseline characteristics

HIS, MOD and CON were comparable (*p* > 0.05) in height, weight, body mass index, systolic blood pressure, diastolic blood pressure, mean arterial pressure, and resting heart rate (Table 1). Baseline age was statistically different between trial groups (*p* = 0.03).

### 3.2 Training responses

Mean and peak heart rates during HIS training were 158 bpm [156; 160] and 176 bpm [175; 177], respectively, corresponding to 85.5% [85.0; 86.0] and 95.3% [94.8; 95.8] of HR_max_, which was higher (*p* < 0.05) than in MOD (132 bpm [130; 134] and 144 bpm [143; 145] equivalent to 72.5% [72.1; 72.9] and 79.1% [78.7; 79.5] of HR_max_).

### 3.3 Cardiac measures at baseline and post‐intervention

Except for left ventricular septum diameter (MOD: 0.88 mm [0.83; 0.93] vs. HIS: 0.77 [0.72; 0.82], *p* = 0.01) and global longitudinal strain (MOD: −20.8 ± 3.4 vs. HIS: −17.8 ± 2.0 and CON: −18.1 ± 2.1, *p* < 0.05), all obtained cardiac measures were similar between the trial groups at baseline. Moreover, all obtained cardiac measures were similar between the trial groups after the intervention except for left ventricular septum diameter (MOD: 0.91 mm [0.86; 0.96] vs. HIS: 0.80 mm [0.75; 0.85] vs. CON: 0.82 mm [0.77; 0.87], *p* < 0.05).

### 3.4 Cardiac adaptations

#### 3.4.1 Left ventricular remodeling

**FIGURE 2:**
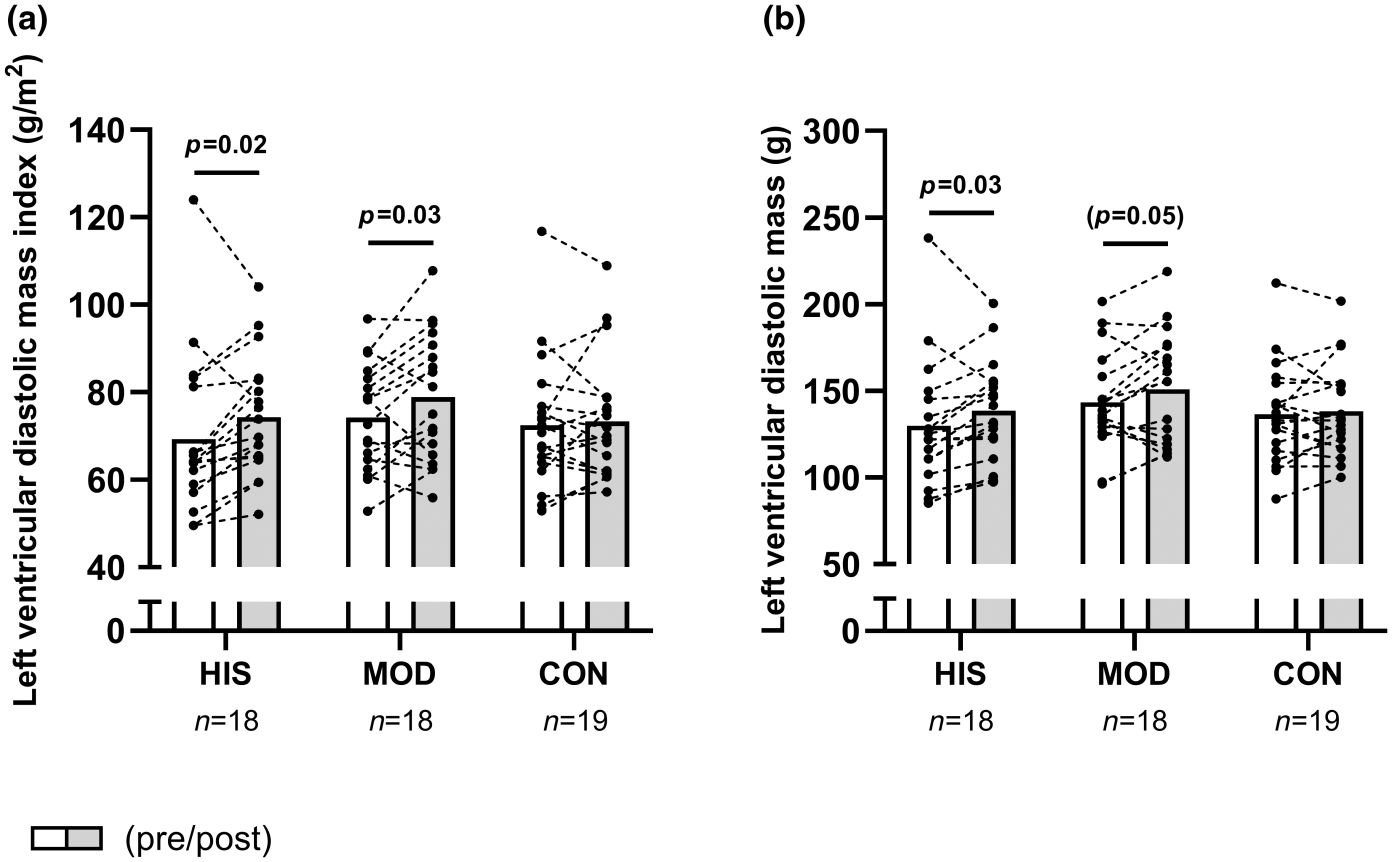
The figure shows mean values for indexed left ventricular mass (a) and left ventricular mass (b) in histograms with each participant shown as a dotted line in HIS, MOD and CON measured before (white bars) and after (gray bars) the 15‐week intervention. *p*‐values from the Sidak‐adjusted pairwise comparisons are presented for significant findings; parentheses indicate statistical tendencies (*p* < 0.1).

High‐ and moderate‐intensity swim training increased left ventricular mass index by 7.3% [1.2; 13.2] (*p* = 0.02) and 6.2% [0.5; 11.8] (*p* = 0.03), whereas no changes were apparent in CON (*p* = 0.67) (Figure 2a), but no statistical between‐group effect existed (time×group, *p* = 0.29). In addition, indexed left ventricular internal dimension at end‐diastole increased (*p* = 0.03) by 2.4% [0.2; 4.6] following MOD only (Table 2). No between‐group or within‐group effect existed for left ventricular septum diameter or left ventricular internal dimension at end‐systole (Table 2).

**TABLE 2:** Cardiac measurements before and after 15 weeks of HIS, MOD, and CON.

Outcome										Time×group *p* value
	Intervention									
	HIS			MOD			CON			
	Pre	Post	*n*	Pre	Post	*n*	PRE	POST	*n*	
Left ventricular adaptations										
IVSd (cm)	0.77 [0.72;0.82]	0.80 [0.75;0.85]	17	0.88 [0.83;0.93]	0.91 [0.86;0.96]	18	0.81 [0.76;0.86]	0.82 [0.77;0.87]	19	0.85
IVSdI (cm/m^2^)	0.41 [0.39;0.44]	0.43 [0.40;0.46]	17	0.46 [0.43;0.48]	0.48 [0.45;0.51]	18	0.43 [0.40;0.46]	0.44 [0.41;0.46]	19	0.75
LVIDd (cm)	4.86 [4.66;5.06]	4.89 [4.69;5.09]	17	4.76 [4.56;4.95]	4.83 [4.64;5.03]	18	4.76 [4.57;4.94]	4.77 [4.59;4.96]	19	0.64
LVIDdI (cm/m^2^)	2.61 [2.49;2.72]	2.64 [2.52;2.75]	17	2.49 [2.38;2.60]	2.55 [2.44;2.66]*	18	2.53 [2.42;2.63]	2.54 [2.43;2.65]	19	0.43
LVIDs (cm)	3.23 [3.04;3.43]	3.14 [2.95;3.33]	15	2.91 [2.73;3.09]	2.99 [2.81;3.18]	17	2.92 [2.74;3.10]	2.88 [2.70;3.06]	17	0.34
LVIDsI (cm/m^2^)	1.73 [1.62;1.84]	1.69 [1.58;1.80]	15	1.53 [1.43;1.64]	1.58 [1.48;1.69]	17	1.56 [1.46;1.66]	1.54 [1.43;1.64]	17	0.32
Right ventricular adaptations										
RVIDd (cm)	2.86 [2.65;3.07]	2.75 [2.54;2.96]	14	2.77 [2.59;2.95]	2.81 [2.63;2.99]	19	2.82 [2.64;3.01]	2.81 [2.63;3.00]	18	0.44
RVIDdI (cm/m^2^)	1.52 [1.41;1.63]	1.47 [1.36;1.58]	14	1.44 [1.34;1.53]	1.47 [1.37;1.57]	19	1.50 [1.41;1.60]	1.50 [1.40;1.59]	18	0.41
TAPSE (cm)	2.33 [2.17;2.48]	2.37 [2.22;2.53]	18	2.47 [2.30;2.64]	2.54 [2.37;2.71]	15	2.34 [2.19;2.49]	2.39 [2.25;2.55]	19	0.97
Diastolic function										
MV E (m/s)	0.70 [0.64;0.77]	0.73 [0.66;0.80]	18	0.72 [0.65;0.78]	0.77 [0.71;0.83] (*)	20	0.77 [0.70;0.83]	0.78 [0.71;0.84]	19	0.54
MV A (m/s)	0.60 [0.53;0.68]	0.52 [0.44;0.59]*	18	0.66 [0.59;0.73]	0.57 [0.50;0.64]**	20	0.60 [0.53;0.67]	0.56 [0.49;0.63]	19	0.52
MV DT (ms)	195 [176;215]	185 [166;205]	18	194 [176;213]	188 [169;206]	20	183 [164;202]	195 [176;214]	19	0.39
E/E' septal	7.82 [6.82;8.82]	8.54 [7.55;9.54]	17	9.02 [8.08;9.96]	9.14 [8.20;10.08]	19	8.78 [7.84;9.72]	9.01 [8.07;9.95]	19	0.74
E/E' lateral	6.00 [5.22;6.77]	6.16 [5.39;6.93]	18	6.66 [5.91;7.42]	7.09 [6.34;7.85](*)	19	6.04 [5.28;6.79]	5.72 [4.96;6.47]	19	0.10
LAd (cm)	3.45 [3.24;3.66]	3.52 [3.31;3.73]	16	3.52 [3.33;3.72]	3.67 [3.48;3.87] (*)	18	3.62 [3.41;3.83]	3.64 [3.43;3.85]	16	0.49
Systolic function										
GLS (%)	−17.8 [−19.1;‐16.5]	−18.3 [−19.6;‐17.0]	15	−20.8 [−22.4;−19.2]	‐19.2 [−20.8;‐17.6]*	10	−18.1 [−19.5;‐16.7]	−19.1 [−20.5;‐17.7]	13	0.02
LVEF (%)	62.7 [59.2;66.1]	65.1 [61.7;68.6]	15	66.8 [63.4;70.1]	68.2 [64.8;71.5]	16	68.0 [64.7;71.3]	69.4 [66.2;72.7]	17	0.92

*Note*: Values are presented as means with 95% confidence intervals. The *p* time×group interaction value of the linear mixed model is presented. The results of the Sidak‐adjusted pairwise comparison is indicated by *p < 0.05, **p < 0.01 compared with pre‐intervention and parentheses (*) indicate a statistical tendency (*p* < 0.09).

Abbreviations: E/E' lateral, ratio of lateral E to E'; E/E' septal, ratio of septal E to E'; GLS, global longitudinal strain; IVDs, left ventricular septum diameter; IVDsI, indexed left ventricular septum diameter; LAd, left atrial diameter; LVEF, left ventricular ejection fraction; LVIDd, left ventricular internal dimension at end‐diastole; LVIDdI, indexed left ventricular internal dimension at end‐diastole; LVIDs, left ventricular internal dimension at end‐systole; LVIDsI, indexed left ventricular internal dimension at end‐systole; MV A, mitral valve A velocity; MV DT, mitral valve deceleration time; MV E, mitral valve E velocity; RVIDd, right ventricular internal dimension at end‐diastole; RVIDdI, indexed right ventricular internal dimension at end‐diastole; TAPSE, tricuspid annulus plane systolic excursion.

#### 3.4.2 Right ventricular remodeling

No statistical between‐group or within‐group effect existed for right ventricular internal dimension at end‐diastole or tricuspid annulus plane systolic excursion (Table 2).

#### 3.4.3 Markers of diastolic function

**FIGURE 3:**
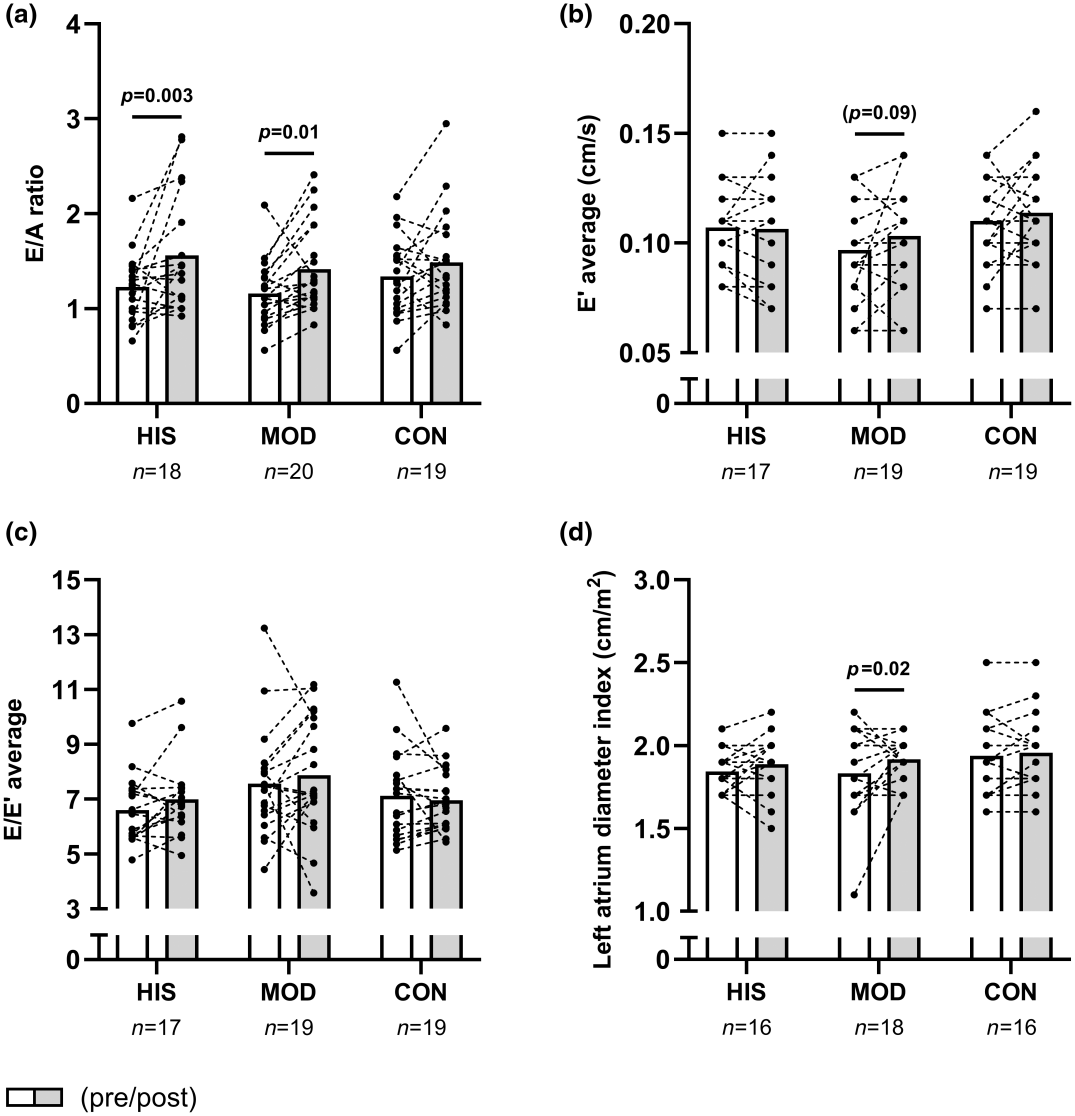
The figure shows mean values for the mitral valve E/A ratio (a), the E' average (b), the E/E' average (c) and the indexed left atrial diameter (d) in histograms with each participant shown as a dotted line in HIS, MOD and CON measured before (white bars) and after (gray bars) the 15‐week intervention. *p*‐values from the Sidak‐adjusted pairwise comparisons are presented for significant findings; parentheses indicate statistical tendencies (*p* < 0.1).

No significant time×group effect existed for any of the obtained markers of diastolic function. However, the pairwise comparison revealed an increased mitral valve E/A ratio of 27% [10; 47] (*p* = 0.003) in HIS and 22% [5; 40] (*p* = 0.01) in MOD, whereas no changes were apparent in CON (Figure 3a). In addition, a borderline significant increase (*p* = 0.09) in the average early diastolic mitral annulus velocity E' of 6.2% [−1; 14] was observed following MOD only (Figure 3b). Furthermore, only MOD demonstrated a borderline significant (*p* = 0.06) increase in mitral valve E velocity of 8% [−0.4; 16]. Conversely, both HIS and MOD resulted in reduced mitral valve A velocity of −14% [−25; −3] (*p* = 0.02) and − 13% [−23; −3] (*p* = 0.01), respectively (Table 2). Finally, left atrial diameter index remained unchanged following HIS and CON, but increased (*p* = 0.02) by 4.9% [0.7; 9.1] following MOD (Figure 3d).

#### 3.4.4 Markers of systolic function

A significant time×group effect (*p* = 0.02) was observed for global longitudinal strain, which appears to be related to a within‐group increase (*p* = 0.03) in global longitudinal strain of 1.6% [0.2; 3.0] increase in MOD, while non‐significant numeric decreases were observed in CON and HIS (Table 2). No between‐group or within‐group effect existed for left ventricular ejection fraction (Table 2).

## 4 DISCUSSION

We conducted a randomized, controlled trial to investigate the effects of a 15‐week high‐intensity swimming protocol and a moderate‐intensity swimming protocol on cardiac structure and function in hypertensive, middle‐aged women using echocardiography. Notably, both high‐ and moderate‐intensity swim training programs resulted in significant within‐group increases in left ventricular mass and improved left ventricular diastolic function indices. However, the magnitude of these changes did not differ between trial groups. Thus, the present findings indicate no clear impact of intensity and/or duration for swim training‐induced adaptations in cardiac structure and function in mildly hypertensive middle‐aged women.

In the present study, both high‐ and moderate‐intensity swim training effectively augmented LV mass to a similar extent, but LV cavity remained unchanged in HIS and slightly increased in MOD, which is consistent with a concentric form of cardiac remodeling. Accordingly, 3 months of endurance‐based running has been shown to induce marked increased in LV mass and mean wall thickness but no effect on LV dimensions in previously sedentary individuals (Arbab‐Zadeh et al., 2014). However, our findings contrast with the slight eccentric cardiac remodeling reported in healthy trained swimmers (mean age of 19 years old) following 12 weeks of intensified swimming (Wasfy et al., 2019). Further, Andersen et al. (2010) demonstrated pronounced increases in LV and RV dimensions following 16 weeks of hybrid training (soccer) in healthy, untrained women (mean age of 38 years old). However, the differences in age, hypertension status, and hormonal profiles between our participants and those in Wasfy et al. (2019) and Andersen et al. (2010) may account for the discrepancies. For instance, evidence has shown that increased age in previously sedentary women has negative impact on cardiac adaptability to endurance‐based exercise training (Carrick‐Ranson et al., 2023; Haykowsky et al., 2005; Spina et al., 1996, 2000). Therefore, direct comparisons should be made cautiously, and our results highlight the need for studies focusing on hypertensive, middle‐aged women. With regards to markers of diastolic and systolic cardiac function, the adaptive response was largely independent of the exercise modality. A significant time×group effect was observed for global longitudinal strain, which arguably relates to the observed within‐group increase in global longitudinal strain after MOD as well as the non‐significant numeric downturn observed after HIS and CON. Global longitudinal strain is a reproducible measure with high prognostic value in various diseases (Lin et al., 2008; Smiseth et al., 2016), but the clinical impact of this finding is unknown. Both exercise groups demonstrated similar within‐group reductions in mitral valve A velocity, hence the mitral valve E/A ratio increased to a similar quantity in both exercise groups. However, only MOD demonstrated a borderline significant within‐group improvement in the average early diastolic mitral annulus velocity, e', a surrogate of ventricular filling pressure (Nagueh et al., 2016). Finally, left atrial diameter, which is highly malleable with exercise training (Mahjoub et al., 2019; Sjúrðarson et al., 2022) increased after MOD only. Thus, it could be speculated that a certain training volume is required to induce changes in e' and left atrial dimension, but the observed effects are trivial, and the long‐term effects are unknown. We acknowledge the importance of considering intervention‐induced changes in blood pressure, given that our study population comprised hypertensive women, and arterial hypertension can independently modify cardiac structure and function. While the present manuscript does not include blood pressure measurements, these data have been published previously (Mohr, Nordsborg, et al., 2014). As reported in Mohr et al. (2014), systolic blood pressure decreased following 15 weeks of both HIS by 6 mmHg (~4%) and MOD by 4 mmHg (~3%), with no statistical difference between the two groups. Conversely, the diastolic blood pressure was unchanged after the intervention in both HIS and MOD. No significant changes were observed in either systolic‐ or diastolic blood pressure in the CON group. Thus, considering the findings from Mohr et al. (2014), which demonstrate similar reductions in blood pressure for both HIS and MOD, it is likely that blood pressure is an unlikely driver of the small differences in cardiac remodeling observed between HIS and MOD in the present study.

Although women reportedly demonstrate a delayed onset of CVD (El Khoudary et al., 2020; Iorga et al., 2017; Kannel et al., 1976; Rosano et al., 2007; Vitale et al., 2009) and CVD has traditionally been considered a men's disease, there are more women than men dying from CVD (Townsend et al., 2022). Numerous CVD risk factors exist that are exclusive to women such as premature menopause, polycystic ovarian syndrome and the menopause (Appelman et al., 2014; Cho et al., 2020; Harvey et al., 2015; Rosano et al., 2007; Sattar & Greer, 2002), which highlights the importance of conducting gender‐specific investigations of risk factor modifying interventions that increase our knowledge of how exercise training duration and intensity impact cardiac adaptability in women. Collectively, the present findings indicate that 15 weeks of swim training induces a concentric form of cardiac remodeling and modestly improves indices of diastolic function in previously untrained, hypertensive women and that these adaptations are principally independent of exercise duration and intensity. Importantly, the length of the training intervention is a significant factor for cardiac adaptability and some evidence suggests that an eccentric form of cardiac adaptations may require up to 6 months to manifest (Arbab‐Zadeh et al., 2014), hence the present training intervention may have been too short to efficiently induce eccentric cardiac remodeling. Finally, an important limitation of our study is the reliance on self‐reported menstrual history without hormonal confirmation of menopausal status. Given the average age of our participants (mean age of ~45 years), it is possible that some were in perimenopausal transition, which may affect hormonal levels, and future studies should include hormonal assessments or utilize standardized classifications like the STRAW+10 criteria (Harlow et al., 2012) to ensure homogeneity in hormonal status.

## 5 STRENGTHS AND LIMITATIONS

We applied a randomized, controlled design, and all training sessions were organized and supervised by trained swim coaches. The trial data were blinded as the echocardiography was performed by one trained cardiologist and the measurements were performed by another trained cardiologist blinded to group allocation and pre/post measurements. However, the study also has a few limitations. First, although our sample was selected after extensive screening of more than 250 women, resulting in homogeneity regarding hypertension status and sedentary lifestyle, we recognize that hormonal status was not uniformly assessed. This limitation may have affected the internal validity of our findings. Second, our trial only lasted 15 weeks, which is a relatively short exercise intervention when assessing cardiac adaptations (Arbab‐Zadeh et al., 2014). Third, the Sidak‐adjusted post‐hoc comparison is considered a conservative test in the adjustment for multiple testing (Shingala & Rajyaguru, 2015), hence an actual cardiac effect might have been missed due to statistical type II errors. Fourth, we solely applied echo and not a combination of echo and cardiovascular magnetic resonance imaging (CMR) as we have previously done (see for example Sjúrðarson et al. (2022)). Echocardiography is currently the first‐line imaging method for the assessment of LV diastolic function (Nagueh et al., 2016, 2019), but CMR has the best inter‐study reproducibility of any non‐invasive imaging techniques for measuring cardiac volume and mass (Bottini et al., 1995; Grothues et al., 2002, 2004). Finally, baseline age was statistically different between trial groups, which may constitute a limitation as some evidence suggests that cardiac adaptability to endurance‐based exercise training is attenuated with increasing age in previously sedentary women (Carrick‐Ranson et al., 2023; Haykowsky et al., 2005; Spina et al., 1996, 2000).

## 6 CONCLUSION

In conclusion, the present findings indicate that 15 weeks of swim training can elicit marked increases in left ventricular mass and enhance markers of left ventricular diastolic function in middle‐aged, pre‐menopausal women with mild hypertension. These changes appear to materialize irrespective of the exercise intensity and duration of the swim training sessions. Thus, both high intensity interval‐based swim training and prolonged continuous moderate intensity swimming seem to initiate beneficial adaptations in cardiac health in middle‐aged, mildly hypertensive, premenopausal women.

## AUTHOR CONTRIBUTIONS

M.M., P.K. and N.B.N. conceived and designed the study. L.J.A. performed the experiments. T.S. analyzed data. T.S. and M.M. interpreted the results of the experiments. T.S. prepared the figures and tables. T.S. and M.M. drafted the manuscript. All authors contributed to writing and reviewing the manuscript and approved the final version of the manuscript.

## FUNDING INFORMATION

The study was supported by the Faroese Research Council, the Faroese Confederation of Sports and Olympic Committee, The Faroese Football Association, Betri Bank and the Danish Sports Confederation.

## CONFLICT OF INTEREST STATEMENT

The authors report no conflict of interest.

## ETHICS STATEMENT

The Ethics Committee of the Faroe Islands approved the study, and all individuals gave written consent prior to participation.

## Data Availability

The datasets generated during and/or analyzed during the current study are available from the corresponding author on reasonable request.
